# Myeloma Spine and Bone Damage Score (MSBDS) on Whole-Body Computed Tomography (WBCT): Multiple Reader Agreement in a Multicenter Reliability Study

**DOI:** 10.3390/diagnostics12081894

**Published:** 2022-08-04

**Authors:** Alberto Stefano Tagliafico, Clarissa Valle, Pietro Andrea Bonaffini, Ali Attieh, Matteo Bauckneht, Liliana Belgioia, Bianca Bignotti, Nicole Brunetti, Alessandro Bonsignore, Enrico Capaccio, Sara De Giorgis, Alessandro Garlaschi, Silvia Morbelli, Federica Rossi, Lorenzo Torri, Simone Caprioli, Simona Tosto, Michele Cea, Alida Dominietto

**Affiliations:** 1 Department of Health Sciences, University of Genoa, 16132 Genoa, Italy; 2 Ospedale Policlinico San Martino, 16132 Genoa, Italy; 3 School of Medicine, University Milano Bicocca, 20126 Milan, Italy; 4 Department of Diagnostic Radiology, Papa Giovanni XXIII Hospital, 24127 Bergamo, Italy; 5 Department of Radiology, Ospedale Santa Corona, 17027 Pietra Ligure, Italy; 6 Department of Vascular Surgery, AOU Pisana, 56124 Pisa, Italy; 7 Department of Internal Medicine, University of Genoa, 16132 Genoa, Italy

**Keywords:** multiple myeloma, computed tomography, quantitative imaging, bone

## Abstract

**Objective:** To assess the reliability of the myeloma spine and bone damage score (MSBDS) across multiple readers with different levels of expertise and from different institutions. **Methods:** A reliability exercise, including 104 data sets of static images and complete CT examinations of patients affected by multiple myeloma (MM), was performed. A complementary imaging atlas provided detailed examples of the MSBDS scores, including low-risk and high-risk lesions. A total of 15 readers testing the MSBDS were evaluated. ICC estimates and their 95% confidence intervals were calculated based on mean rating (k = 15), absolute agreement, a two-way random-effects model and Cronbach’s alpha. **Results**: Overall, the ICC correlation coefficient was 0.87 (95% confidence interval: 0.79–0.92), and the Cronbach’s alpha was 0.93 (95% confidence interval: 0.94–0.97). Global inter- and intra-observer agreement among the 15 readers with scores below or equal to 6 points and scores above 6 points were 0.81 (95% C.I.: 0.72–0.86) and 0.94 (95% C.I.:0.91–0.98), respectively. **Conclusion:** We present a consensus-based semiquantitative scoring systems for CT in MM with a complementary CT imaging atlas including detailed examples of relevant scoring techniques. We found substantial agreement among readers with different levels of experience, thereby supporting the role of the MSBDS for possible large-scale applications. **Significance and Innovations** • Based on previous work and definitions of the MSBDS, we present real-life reliability data for quantitative bone damage assessment in multiple myeloma (MM) patients on CT. • In this study, reliability for the MSBDS, which was tested on 15 readers with different levels of expertise and from different institutions, was shown to be moderate to excellent. • The complementary CT imaging atlas is expected to enhance unified interpretations of the MSBDS between different professionals dealing with MM patients in their routine clinical practice.

## 1. Introduction

In multiple myeloma (MM), there is an abnormal and excessive production of monoclonal immunoglobulin M derived from plasma cells. The pathological alteration of bone marrow, shown in both histopathology and medical imaging, is due to the increased presence of plasma cells, which is the main feature of MM [1,2,3,4,5,6]. In addition, in MM, there is an imbalance in the activation of osteoclasts and osteoblasts often, but not only, derived from a single tumoral clone. Medical imaging, such as computed tomography (CT), is normally used to detect bone disease in MM, which is responsible for a reduction in quality of life mainly due to pain and pathological fractures. Bone disease is also related to increased mortality. In addition, new therapies for MM are going to be developed and tested in clinical trials, where imaging techniques can be considered as a surrogate endpoint, especially in patients with early disease [2,3,6,7,8,9,10]. Alongside these developments in medical therapies, imaging tools have been showing progressive improvements over the last decades, with increased complexity. Indeed, imaging is clearly crucial in the diagnosis, management, and follow-up of patients with MM, as has been reported in the most recent guidelines [1,5,6,9,11]. Due to these developments, numerous randomized controlled trials on disease-modifying drugs and other treatment strategies for MM are expected to be implemented or are already under preparation. The use of CT for MM is widespread in most oncological centers, is readily available and is amenable to second-look evaluations in order to increase reliability [12]. Therefore, CT-based scoring systems could be suitable instruments for use in clinical trials if reliability is assured. Unfortunately, there is little or even no consensus yet on CT scoring systems, even with regards to the most elementary MM lesions [1,8,12]. In 2020, a preliminary CT-based scoring system, called the “*myeloma spine and bone damage score*” (MSBDS), was developed and included semi-quantitative assessments of elementary lesions using descriptive criteria with the aim of harmonizing total body CT interpretation in MM [13]. Despite the good results obtained by the authors, it should be noted that this scoring system was tested by a limited number of readers [3] on a small series of patients. Therefore, the aim of the current study was to test the reliability and the inter- and intra-observer agreement of the MSBDS with multiple readers with different levels of expertise and from different institutions in order to simulate the real-life application of this system. 

## 2. Methods

### 2.1. Patients

Approval from the institutional review board was obtained (054REG2019). The patients involved in this study provided written informed consent for this retrospective research before CT examinations were performed. Due to the nature of this study, data analysis for this study was not used to influence patient care. CT examinations were acquired with different CT scanners: (1) two 128-slice scanners (Siemens SOMATOM Definition Flash), using the following parameters adjusted according to patients’ characteristics: collimation 64 × 0.6 mm; spatial resolution: 0.30 mm; scanning time: 36 s; length of scanning: 650 mm; rotating time: 0.33 s; tube current: 100/140 kV, 50/60 mAs; effective dose: 2.6 mSv, (2) one 64-slice scanner (GE Optima 660) and (3) one 16-slice scanner (GE Lightspeed). Of the 150 consecutive patients (mean age, 59 years; range, 35–79 years; 72 females; 78 males) admitted to the hospital (BLIND for REVIEW) in the last three years, because of suspicions of MM that were later confirmed by bone biopsy, anonymized images and complete CT examinations were collected from 104 patients with relevant bone abnormalities (diffuse pattern or focal lesions). This pool of 104 patients with complete CT examinations was selected by two authors (who were not involved in the reading sessions) to represent all degrees of pathology related to the osseous involvement of MM, including localizations with extraosseous extension. The proportion of CT images acquired using different scanners was balanced across all of the images that were evaluated in this study: 30% of the images were obtained using the 128-slice CT, 30% with the 64-slice CT and 40% with the 16-slice CT. A lytic lesion, in both axial and extra-axial skeletons, had to be >5 mm in diameter to be considered significant, as per the most recent guidelines [2,4,5,6,7,11,12,13]. Patient risk stratification was performed using the Revised International Staging System (ISS), combining serum beta2-microglobulin and serum albumin, lactate dehydrogenase for three-stage classification and cytogenetics to determine a binary normal–high risk stadiation [5,6,7].

### 2.2. CT Imaging Atlas

A preliminary CT atlas was developed for use in the web-based evaluation (see further) and was edited according to evaluations made by three radiologists with expertise in musculoskeletal imaging (F.R, B.B and C.V. with 5, 6 and 7 years of experience, respectively). Images were collected from the inpatients who underwent a whole-body CT at the university and hospital Hematologic Unit. After collection, the CT images were anonymized for the web-based evaluation.

### 2.3. Web-Based Evaluation 

A web-based reliability study was performed using the CT atlas containing a pool of 104 CT exams that were selected by a radiologist with 15 years of experience in musculoskeletal radiology (A.T.) who was not involved in the MSBDS assessment. The CT images and exams were selected to have a balanced degree of pathology involving the bones and the adjacent tissue. For the inter-reader reliability assessment, the MSBDS was assigned to every item available to readers. Subsequently, for intra-reader reliability, 44 images were randomly chosen and redistributed two months after the first round in order to guarantee sufficient wash-out. In this way, each reader evaluated a reduced sample of CT images that were randomly selected from the previous patient’s cohort. MSBDS is a semiquantitative additive scale where the total score is the sum of the single items related to the abnormalities detected. MSBDS values range from 0 (minimum) to values >10, where 10 or more is represented by high-risk patients requiring immediate surgical or radiation oncologist evaluation [13]. The MSBDS scoring systems are briefly recalled in Table 1. In this study, 15 readers were selected from different institutions and with different expertise, not only regarding their expertise in bone evaluation using CT images, but also regarding their medical specializations. Among these readers, there were eleven radiologists (three of whom subspecialized in musculoskeletal radiology), one vascular surgeon, one radiation oncologist, one expert in legal medicine and one hematologist. An expert with 15 years of experience in musculoskeletal radiology (with a strong track record and a diploma from the European Society of Musculoskeletal Radiology) led a training session for every reader before they commenced the web-based reliability exercise, a number of whom were present for the training (10/15 readers) and a number completed the training remotely (5/15 readers).

### 2.4. Statistical Analysis

As a measure of MSBDS reliability for both the degree of correlation and agreement between measurements, the intraclass correlation coefficient (ICC) was calculated as an index [14]. The aim of this paper was to generalize the results from our reliability exercise to any raters or readers who possess the same characteristics of expertise as the selected raters in this reliability study. Therefore, we used a two-way random-effects model according to Shoukri [15] MM et al. [15]. As a commonly suggested rule of thumb, we obtained far more than 30 heterogeneous samples (104 samples in this study) and involved more than 3 raters (15 raters) to conduct this reliability study. ICC values that were less than 0.5 were considered indicative of poor reliability. ICC values between 0.5 and 0.75 indicated moderate reliability, whereas ICC values between 0.75 and 0.9 indicated good reliability. Finally, ICC values greater than 0.90 indicated excellent reliability [16]. In this study, ICC estimates and their 95% confidence intervals were calculated based on a mean rating (k = 15), absolute agreement and a two-way random-effects model. Cronbach’s alpha was used to assess the internal consistency of the method; values of alpha that were above or equal to 0.90 were considered useful for clinical purposes. Statistical analyses were performed using software for Windows (MedCalc—version 12.3.0) [17]. 

## 3. Results

The main characteristics of the 104 MM patients enrolled in the study, including clinical data, are shown in Table 2.

### 3.1. CT Imaging Atlas

**Figure 1:**
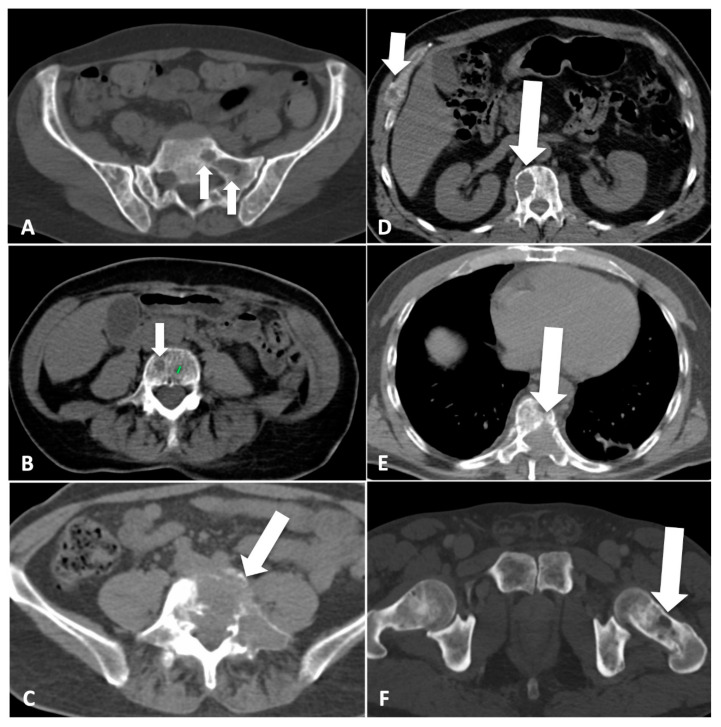
Scoring bone damage and instability: spectrum of findings. (**A**) Focal lytic lesions >5 mm in diameter located at the left sacrum (white arrows). In this case the MSBDS was 2 (1 + 1). (**B**) Single focal lytic lesion >5 mm in the vertebral body (white arrow) with no vertebral collapse (sagittal not shown). The adjacent smaller focal lytic lesion (green line) is <5 mm (no points in the MSBDS). In this case, the MSBDS was 1. (**C**) Large lytic lesion at the junctional spine (L5-S1) with cortical erosion, collapse/involvement >50%, posterolateral (facet, pedicle) involvement and more than 2/3 of bone diameter. In this case, the MSBDS was 11 (3 + 3 + 2 + 3): the lesion was considered “high-risk” and immediate surgical or radiation oncologist consultation was warranted. In this case, there was also possible spinal canal involvement. (**D**) Lytic lesion >5 mm (white arrow) at the junctional spine (thoracic spine) with collapse/involvement <50% and a small (small white arrow) focal lesion at the anterior arch of the right rib cage with extraosseous extension. In this case, the MSBDS was 6 (3 + 2 + 1): the lesion was considered “medium-risk” (5–10 with medium risk of pathologic fracture). (**E**) Large lytic lesion at the junctional spine (thoracic spine) with collapse/involvement >50%, posterolateral (facet, pedicle) involvement and more than 2/3 of bone diameter. In this case, the MSBDS was 11 (3 + 3 + 2 + 3): the lesion was considered “high-risk” and immediate surgical or radiation oncologist consultation was warranted. In this case, there is spinal canal involvement. (**F**) Lytic lesion at the left femoral neck (white arrow). This lesion alone warrants 5 points in the MSBDS: the lesion was considered “medium-risk”, although immediate fracture seems unlikely.

A CT imaging atlas of lesions that were detectable using the MSBDS was prepared, including a comprehensive version that was available for the reliability assessment, and was prepared using a complete spectrum of lesions broad enough to give coverage of all possible lesions, ranging from small lytic (<5 mm), diffuse patterns to large lesions with destructive and extra-medullary patterns (Figure 1). 

### 3.2. Web-Based Reliability Assessment

**Figure 2:**
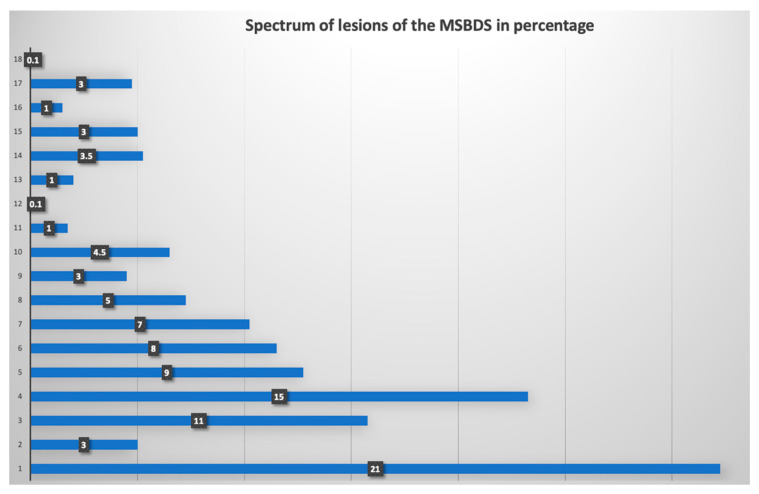
Frequency of bone lesions in the web-based reliability assessment. In this graph, the distribution of the degree of pathological findings, according to the MSBDS, is presented. The range of the MSBDSs was between 1 and 18. Most lesions (21%) were small lesions with an MSBDS of 1.

Overall, the 15 readers completed the web-based reliability assessment of the scoring systems. The staging and the spectrum of bone lesions were sufficiently broad in order to give acceptable coverage of the severity of the disease as reported in the staging system. The distribution of the degree of pathological findings according to the MSBDS is reported in Figure 2. The coefficient of variation of the lesions analyzed was 0.8, the mean score was 5.5 and the interquartile range (25–75) was 6. The entire spectrum of possible bone disease, according to the MSBDS, was present, but there was a low prevalence of the highest score, thus reflecting the peculiar robustness of the method and the possible clinical impact, particularly in the lower stages. The mean time to perform the analysis was 2 min per patient. 

### 3.3. Overall Agreement for MSBDS

**Figure 3:**
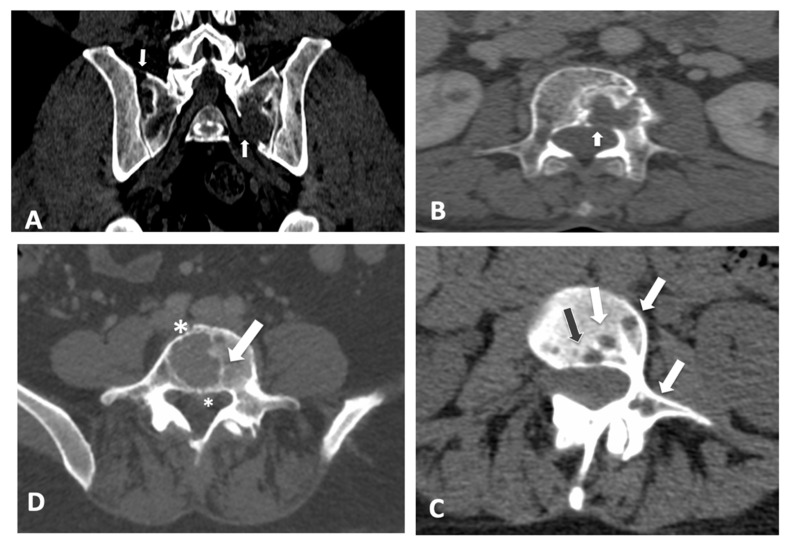
Scoring bone damage and instability: spectrum of findings with maximum disagreement among readers. Discrepancies with an MSBDS > 6. (**A**) Focal lytic lesions >5 mm in diameter located at the left sacrum (white arrow) with another small lesion in the sacrum near the sacroiliac joint. In this case, the MSBDS was 7 with a standard deviation of 4.9. (**B**) Single large focal lytic lesion >5 mm in the vertebral body (white arrow) with no vertebral collapse (sagittal not shown) but possible spinal canal infiltration. In this case, the MSBDS was 11 with a standard deviation of 5.3 due to difficulties in spinal canal assessment mainly by non-specialists. In these cases, a sub-specialized second reading should be recommended. Discrepancies with an MSBDS < 6. (**C**) Multiple lytic lesions >5 mm (white arrows and the black arrow) at the junctional spine (T11-L1 level) with involvement of the vertebral body and pedicle. In this case, the MSBDS was 4 with a standard deviation of 2.6. (**D**) Single large focal lytic lesion >5 mm in the vertebral body (white arrow) with no vertebral collapse. In this case, the MSBDS was 5.7 with a standard deviation of 3.3 due to difficulties in spinal canal assessment, bone diameter and extraosseous involvement (asterisks).

Overall, the ICC correlation coefficient was 0.87 (95% confidence interval: 0.79–0.92), reflecting the degree of consistency among measurements. The ICC for the single measure was 0.69 (0.61–0.77). Overall, Cronbach’s alpha was 0.93 (95% confidence interval: 0.94–0.97). The inter-reader agreement was 0.68 (0.56–0.79) with a standard error of 0.05. Global inter- and intra-observer agreement among the 15 readers considering the MSBDS with scores below or equal to 6 points and scores above 6 points are reported in Table 3. Some examples of images where maximal discrepancies were found are reported in Figure 3. The ICC was not influenced by different CT scanners. 

## 4. Discussion

In this study, we developed and assessed a consensus-based scoring system for MM bone lesions detectable with CT using a previously validated methodology [18,19].

Based on previous CT evaluations of MM-related lesions, we scored the clinically relevant lytic lesions, which are >5 mm in diameter, with the aim of creating a simple, reliable, reproducible and semiquantitative method consistent in radiological reporting [13]. This scoring system is a novel scoring system of bone lesions tailored specifically for use in MM with the purpose of being used with standard whole-body CT in daily clinical practice and to complement common evaluations. Indeed, bone damage is a typical target area in patients who are affected by MM, and numerous trials investigating potential structure-modifying or bone-protective treatments and other management strategies for MM are anticipated. Our scoring system may be an instrument for the domains determined by the Revised International Staging System. Bone lesions, such as lytic lesions detected by radiography, CT or PET/CT, are the myeloma-defining events belonging to the standard CRAB criteria (calcium elevation, renal failure, anemia, lytic bone lesions) [6,20]. In MM patients, imaging the bone is crucial because the detection of an osteolytic lesion provides a reason to treat. In patients with confirmed MM, medical imaging can be used to localize the source of pain and to prevent any eventual complications that are usually related to bone destruction [21]. The focal lesions detected on medical imaging are a strong prognostic marker and mark the need for treatment, even in the absence of cortical bone destruction [22,23,24]. Despite the diagnostic value of CT for the detection of bone marrow lesions, it is limited and MRI is superior. However, the possibility to use a reliable score to evaluate computed tomography images with acceptable agreement among different readers, such as the MSBDS, could be clinically helpful. In addition, the complementary CT imaging atlas is expected to enhance unified interpretations of the grading scales between radiologists and clinicians across countries and departments. Indeed, MM-associated bone disease impacts quality of life, with increases in both morbidity and mortality [3,25,26,27,28,29]. CT, as well MRI, is important when diagnosing MM, especially in the detection of bone lesions when they are small and when they are amenable to intervention, even to prevent pathological fractures or neurological complications due to the compression of the medulla or nerves [3,5,7,10,25,26,27,28,29,30,31,32]. CT-proven MM lesions were tested in a web-based exercise with substantial to excellent agreement. The CT images selected for the web-based exercises were of high quality, were pre-selected and did not include image selection and acquisition. The current semiquantitative score may be more helpful in clarifying the role of CT in MM bone and bone marrow imaging compared to the standard reporting of CT imaging, which is improved by sub-specialized second-opinion reading [12]. Indeed, in MM CT evaluation, discrepancies when interpreting a clinically important abnormality, such as the presence of a lytic lesion >5 mm, could be present in up to 21% of patients and has the potential to impact treatment planning [12]. The database was comprised mostly of stage I and II lesions, and most of them (22%) were small lesions (MSBDS 1), as is expected in normal clinical practice. Some variation in intra-class reliability is probably due to initial difficulty in applying new definitions of the ‘real life’ application of anatomical terms which are not so defined when dealing with 5 mm lesions. For example, it is difficult to clearly define the borders or limits of a posterolateral facet or pedicle involvement, the limit of spinal canal involvement or the “entire” trochanteric region, especially when the readers are not radiologists involved in medical imaging assessment and reporting on a daily basis. To reduce further variability when reading CT images, we believe that appropriate training could be useful, but, when possible, double reading of CT images could be the best choice, even for lesion identification. In this study, the MSBDS scoring system had a high concordance among many readers. The number of readers involved in this study and their different levels of expertise is very high, which is a strength of the current analysis. In addition, the scoring system was fast and suitable for routine clinical practice, and can be applied in clinical practice when reading or reporting CT in patients with MM. As opposed to the first application of the MSBDS, which was performed on a relatively high number of patients with advanced stages of disease for demonstrative and academic applications [13], in this work, the majority of lesions (22%) were small lesions with an MSBDS of 1, and we aimed to enhance the clinical utility of the scoring systems. Indeed, at least in theory, it is very important to correctly stratify patients at early stage of disease. We have to remember that MSBDS is strongly correlated with the Myeloma Response Assessment and Diagnosis System (MY-RADS) [13], confirming the relevance of the MSBDS scoring system [33,34,35,36]. The MSBDS is suitable for CT images, is typically more available than MRI and has the potential to be highly applicable for large scale clinical trials with imaging parameters as endpoints. The prospective clinical validation of MSBDS criteria regarding its clinical impact on patients is underway.

In conclusion, we presented a consensus-based semiquantitative scoring system for CT in MM together with a complementary CT imaging atlas, in which detailed examples of relevant scoring techniques are provided. We found substantial agreement among readers with different levels of experience, supporting the role of MSBDS for large scale applications. MSBDS is specifically tailored for use with patients who are affected by MM and differs from other scoring systems that have been developed in orthopedic literature for bone metastasis [37]. 

## Figures and Tables

**Table 1:** MSBDS (Myeloma Spine and Bone Damage Score). Interpretation: High-risk: >10, requiring immediate surgical or radiation oncologist consultation. Medium risk: ≥5–10, possible instability and a medium risk of pathological fracture. Low-risk: <5. * Bone abnormalities not sufficient to give high-risk scores, if isolated. ** 1 point for every segment according to MY-RADS [13].

Location	Points
Junctional Spine (C0-C2, C7-T2, T11-L1, L5-S1)	3
Mobile Spine (C3-C6, L2-L4) * only 1 point for semi-rigid (T3-T10)	2
Collapse/involvement >50%	3
Collapse <50% *	2
Posterolateral (facet, pedicle) involvement monolateral	2
Posterolateral (facet, pedicle) bilateral monolateral	3
Spinal Canal involvement	5
Trochanteric region focal lesions <10 mm	2
Femoral neck or entire trochanteric region	5
More than 2/3 of bone diameter	3
Focal lesion >5 mm at any site *	1
Diffuse Pattern	1 **

**Table 2:** Clinical data of the 104 MM patients included in the study. High-risk defines MM patients carrying HR features, including del17p, t(4;14) or t(14,16), according to the FISH analysis. International Staging System includes stage I-III based on beta2-microglobulin and albumin levels.

	Number	Percentage
Patients	104	100
Age (mean years)	58	
Age Standard Deviation	8.1	
Males	62	59.6
Females	42	40.4
**Cytogenetics**		
Normal	72	69.2
High-risk	32	30.8
**Relapsed**		
	71/104	68
Days before relapse (mean)	1173	
Days of follow-up (mean)	1466	
**International Staging System**		
Stage I	48	46
Stage II	28	27
Stage III	28	27

**Table 3:** Global inter- and intra-observer agreement among the 15 readers considering the MSBDS with scores below or equal to 6 points and scores above 6 points. K values are reported as weighed with linear weights.

**Inter-observer**	***ICC***	**95% Confidence Interval**
MSBDS ≤ 6 points	0.81	0.72–0.86
**Intra-observer**	***ICC***	**95% Confidence Interval**
MSBDS ≥ 6 points	0.94	0.91–0.98

## Data Availability

The datasets generated during and/or analyzed during the current study are available from the corresponding author on reasonable request.

## Abbreviations

MM	multiple myeloma
CT	computed tomography
ISS	Revised International Staging System
ICC	intraclass correlation coefficient
MSBDS	Myeloma Spine and Bone Damage Score
MRI	magnetic resonance imaging
MY-RADS	Myeloma Response Assessment and Diagnosis System
